# Supplementary material for: Evidence of Neutrophils and Neutrophil Extracellular Traps in Human NMSC with Regard to Clinical Risk Factors, Ulceration and CD8+ T Cell Infiltrate
Source: Int J Mol Sci. 2024 Oct 2;25(19):10620. doi: 10.3390/ijms251910620 (PMC11476888; doi:10.3390/ijms251910620)
Supplement: Supplementary file 1 [file ijms-25-10620-s001.zip › FigureS1.pdf]

A

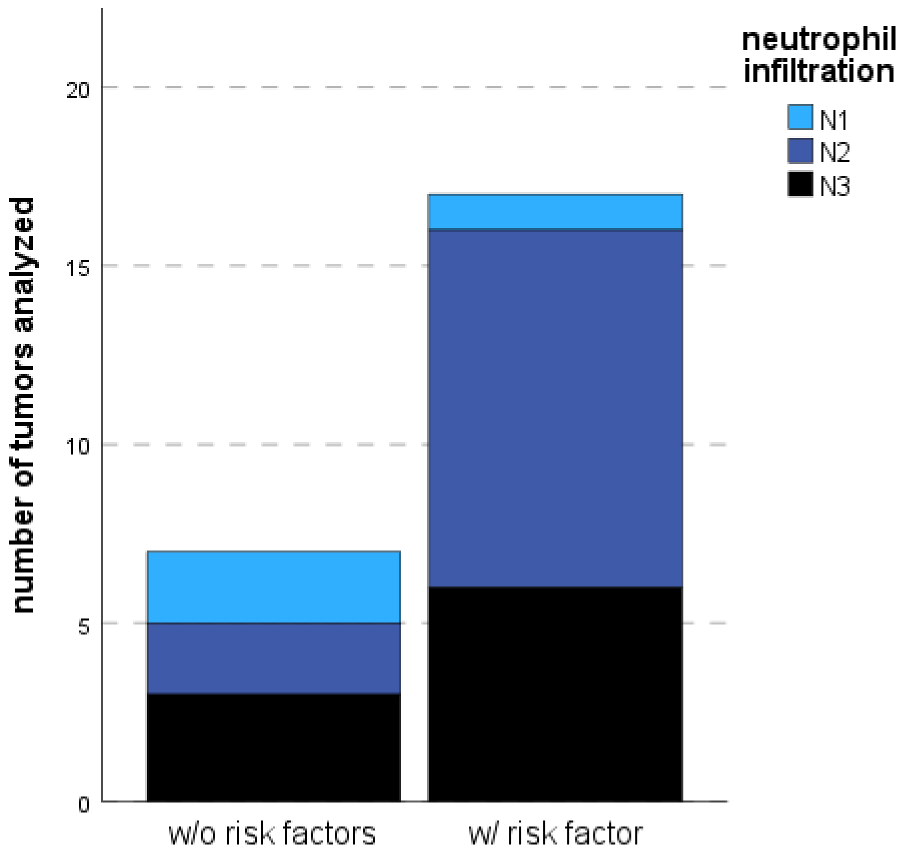

B

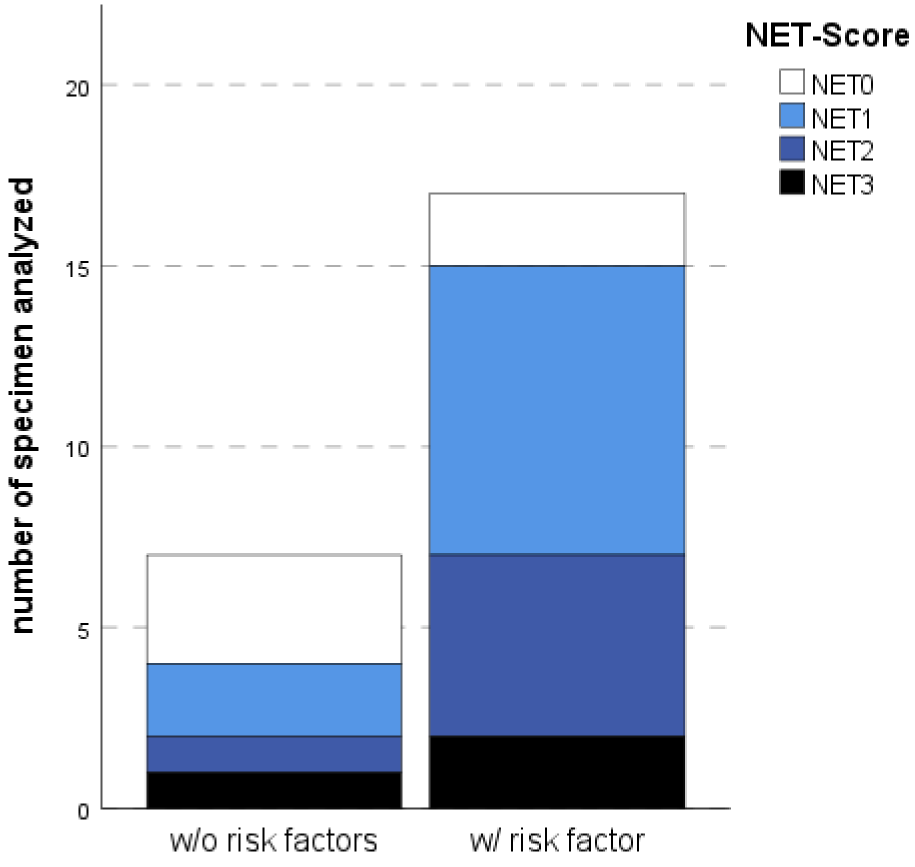

Figure S1:

(**A**) Neutrophil infiltration did not differ significantly between cSCC tumors with one or more of the risk factors immunosuppression, invasion depth ≥ 6mm or grading ≥ G3 and those without (p = .8042). (**B**) Same applied to NET infiltration (p = .3176).
