# Supplementary material for: Evidence of Neutrophils and Neutrophil Extracellular Traps in Human NMSC with Regard to Clinical Risk Factors, Ulceration and CD8+ T Cell Infiltrate
Source: Int J Mol Sci. 2024 Oct 2;25(19):10620. doi: 10.3390/ijms251910620 (PMC11476888; doi:10.3390/ijms251910620)
Supplement: Supplementary file 1 [file ijms-25-10620-s001.zip › FigureS2.pdf]

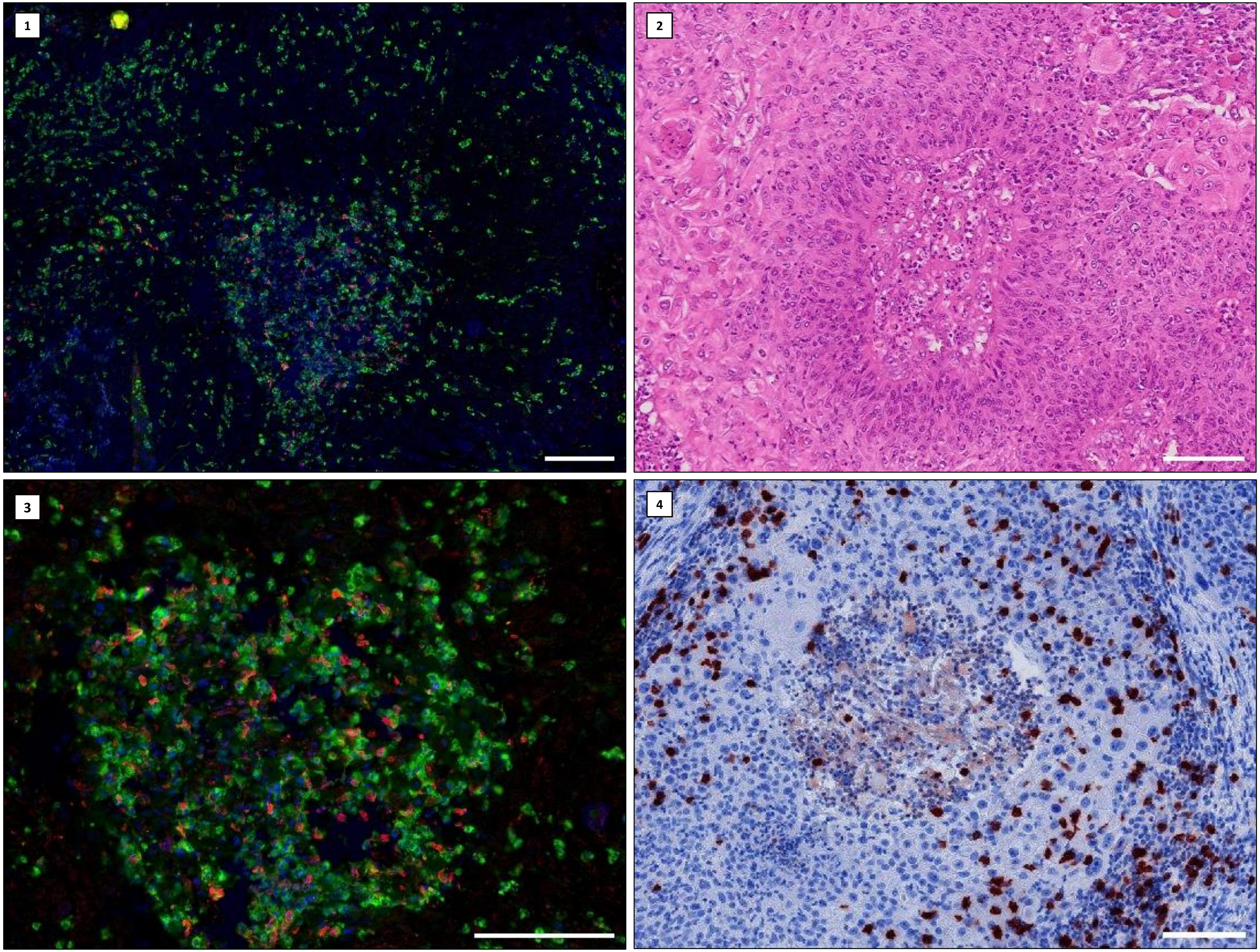

Figure S2:

In cSCC, neutrophil swarming areas and abscess-like structures could be observed. Immunofluorescence staining in 10x (1) and 20x (3) magnification of a neutrophil (CD15, green) swarming area with NETs (H3cit, red). DAPI, depicted in blue, served as a counterstaining for nuclei. This phenomenon could be seen also in the H&E-stained slides (2) and immunohistochemistry (4) for $CD8^{+}$ T cells (brown) that generally did not enter these structures. Scale bar 100 µm.
