# Supplementary material for: Evidence of Neutrophils and Neutrophil Extracellular Traps in Human NMSC with Regard to Clinical Risk Factors, Ulceration and CD8+ T Cell Infiltrate
Source: Int J Mol Sci. 2024 Oct 2;25(19):10620. doi: 10.3390/ijms251910620 (PMC11476888; doi:10.3390/ijms251910620)
Supplement: Supplementary file 1 [file ijms-25-10620-s001.zip › FigureS3.pdf]

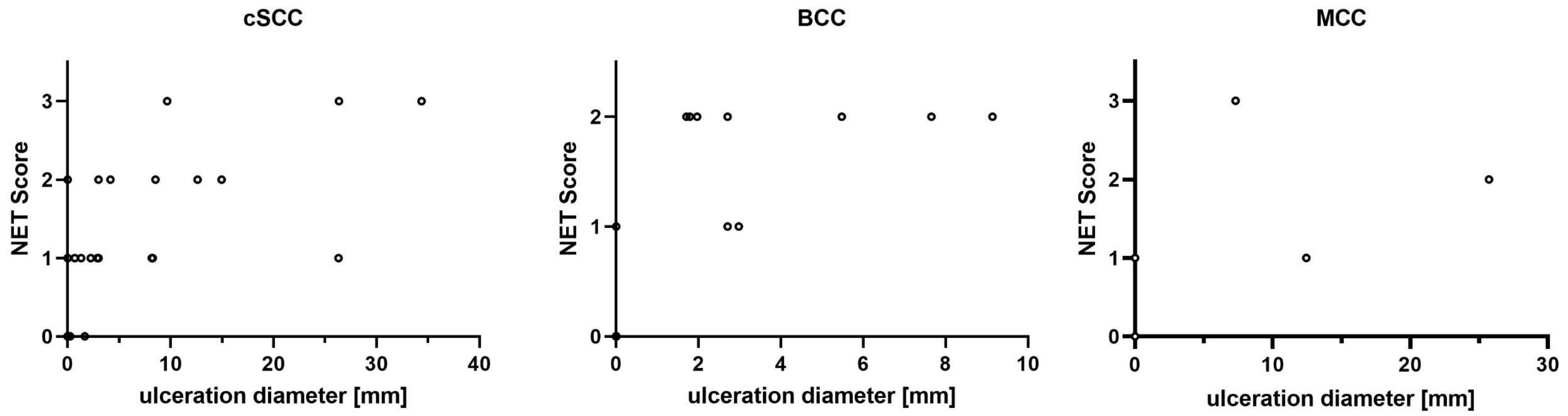

Figure S3:

In all analyzed tumor entities, a positive correlation between ulceration diameter and NET infiltration was observed (Spearman's ρ = .685, two-tailed $p < .001$ for cSCC, Spearman's ρ = .825, two-tailed $p < .001$ for BCC, Spearman's ρ = .843, two-tailed p = .0061 for MCC).
