# Supplementary material for: Evidence of Neutrophils and Neutrophil Extracellular Traps in Human NMSC with Regard to Clinical Risk Factors, Ulceration and CD8+ T Cell Infiltrate
Source: Int J Mol Sci. 2024 Oct 2;25(19):10620. doi: 10.3390/ijms251910620 (PMC11476888; doi:10.3390/ijms251910620)
Supplement: Supplementary file 1 [file ijms-25-10620-s001.zip › FigureS4.pdf]

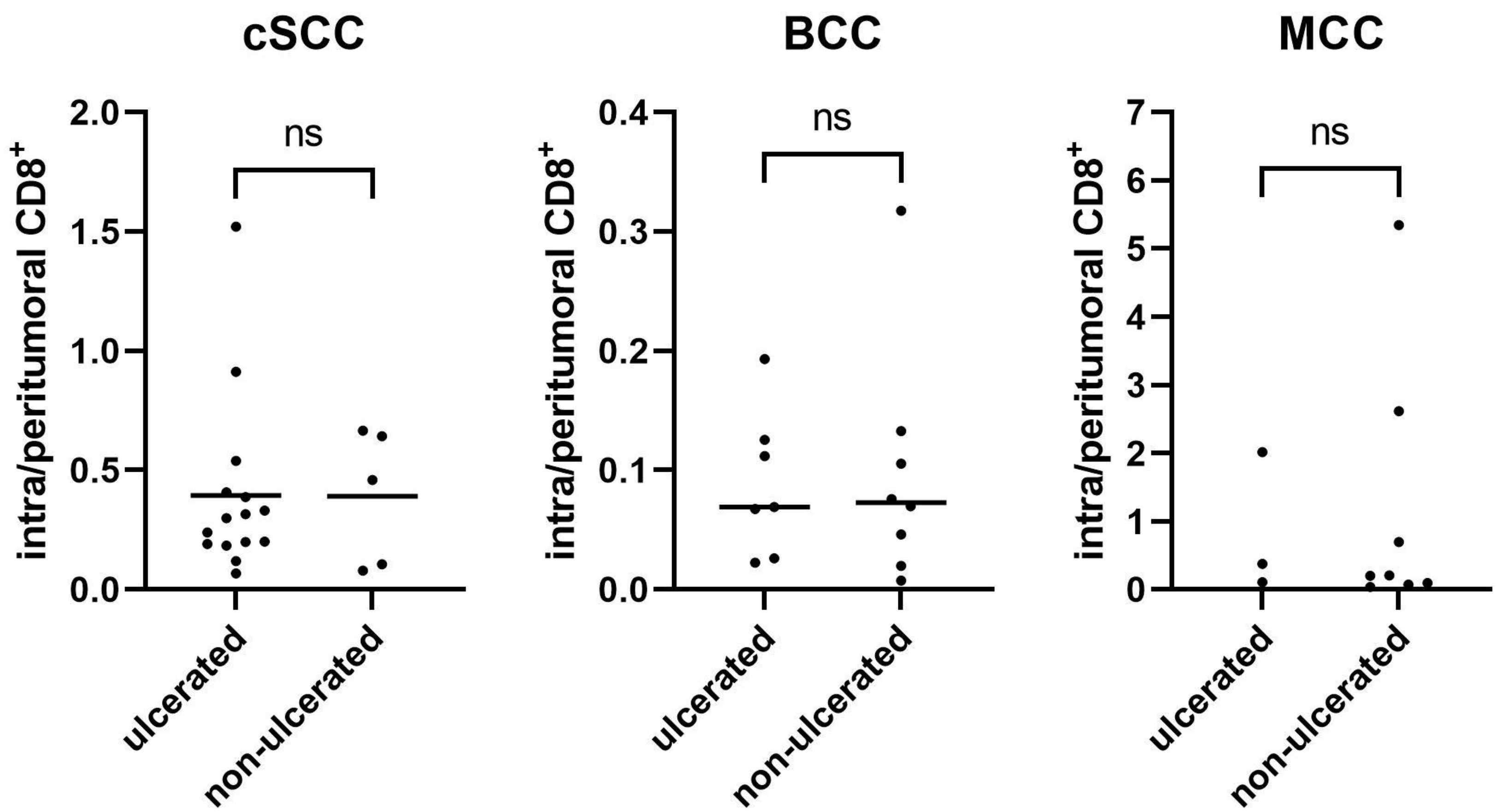

Figure S4:
In no tumor entity a significant difference in the density quotient between ulcerated and non-ulcerated tumors could be seen.
