# Supplementary material for: Evidence of Neutrophils and Neutrophil Extracellular Traps in Human NMSC with Regard to Clinical Risk Factors, Ulceration and CD8+ T Cell Infiltrate
Source: Int J Mol Sci. 2024 Oct 2;25(19):10620. doi: 10.3390/ijms251910620 (PMC11476888; doi:10.3390/ijms251910620)
Supplement: Supplementary file 1 [file ijms-25-10620-s001.zip › Table S1.pdf]

Table S1: Overview of primary tumors

tumor type	sample	patient	sex	age [years]	ulcerated	ulceration diam. [mm]	maximal diam. [mm]	CSA [mm^2]	clinical risk factor	neutrophil score [0-3]	NET score [0-3]	CD8$^+$ intratumoral [cells/mm^2]	CD8$^+$ peritumoral [cells/mm^2]	CD8$^+$ quotient intra-/peritumoral[%]
BCC	1	A	m	77,1	no	0,00	n.a.	0,67	n.a.	0	0	32,02	457,43	7,00
BCC	2	B	f	68,8	no	0,00	n.a.	0,91	n.a.	1	0	24,35	528,80	4,60
BCC	3	C	m	69,9	no	0,00	n.a.	6,12	n.a.	1	1	4,60	619,41	0,74
BCC	4	D	f	88,4	no	0,00	n.a.	0,49	n.a.	1	0	18,15	922,40	1,97
BCC	5	E	m	71,8	yes	2,98	n.a.	1,67	n.a.	1	1	92,24	735,42	12,54
BCC	6	E	m	71,8	no	0,00	n.a.	0,54	n.a.	1	0	56,32	742,84	7,58
BCC	7	F	f	68,3	yes	5,48	n.a.	7,78	n.a.	2	2	9,48	360,65	2,63
BCC	8	G	f	81,2	yes	9,14	n.a.	2,30	n.a.	2	2	53,03	783,94	6,76
BCC	9	H	m	81,8	no	0,00	n.a.	1,03	n.a.	0	0	49,66	156,38	31,76
BCC	10	I	m	78,1	yes	1,79	n.a.	5,95	n.a.	2	2	62,67	905,19	6,92
BCC	11	J	f	67,5	no	0,00	n.a.	0,92	n.a.	0	0	19,67	186,21	10,56
BCC	12	K	m	73,6	yes	2,71	n.a.	2,68	n.a.	2	2	161,67	837,59	19,30
BCC	13	L	m	47,3	yes	1,71	n.a.	12,60	n.a.	2	2	23,07	206,01	11,20
BCC	14	M	f	83,3	yes	7,66	n.a.	8,46	n.a.	2	2	n.e.	n.e.	n.e.
BCC	15	N	m	79,9	yes	1,97	n.a.	1,83	n.a.	2	2	11,64	517,56	2,25
BCC	16	O	m	69,7	yes	2,71	n.a.	12,96	n.a.	2	1	n.e.	n.e.	n.e.
BCC	17	P	m	72,7	no	0,00	n.a.	16,06	n.a.	1	0	112,37	846,32	13,28
cSCC	18	Q	m	75,7	yes	4,17	21,13	139,93	D	2	2	112,15	1657,70	6,77
cSCC	19	R	m	60,6	yes	0,69	3,75	7,94	I	1	1	104,51	567,10	18,43
cSCC	20	M	f	68,7	yes	34,40	23,19	176,63	G, D	3	3	n.e.	n.e.	n.e.
cSCC	21	O	m	70,4	yes	3,01	10,86	58,41	G, D	2	1	13,65	35,20	38,78
cSCC	22	S	m	76,4	yes	3,01	5,74	10,26	G	2	2	346,31	1094,10	31,65
cSCC	23	T	m	73,1	yes	0,25	3,97	3,63	none	1	0	348,05	853,56	40,78
cSCC	24	U	f	80,7	no	0,00	4,87	4,75	none	1	0	n.e.	n.e.	n.e.
cSCC	25	V	m	77,5	no	0,00	15,51	72,89	none	3	1	552,63	1201,70	45,99
cSCC	26	W	m	89,8	yes	9,68	25,86	142,82	none	2	3	225,78	1905,50	11,85
cSCC	27	X	m	69,9	yes	26,36	22,25	110,39	I	3	3	n.e.	n.e.	n.e.
cSCC	28	Y	m	79,5	yes	2,89	9,77	15,23	I	3	1	465,09	509,81	91,23
cSCC	29	Z	m	83,7	yes	26,33	25,83	83,04	G	2	1	230,67	965,46	23,89
cSCC	30	AA	m	92,7	yes	8,56	12,63	48,74	G	2	2	1078,00	708,83	152,08
cSCC	31	BB	m	76,7	yes	1,69	9,17	27,84	G, I	2	0	310,00	575,81	53,84
cSCC	32	CC	m	83,2	yes	12,62	8,98	30,97	none	3	2	55,37	289,23	19,14
cSCC	33	DD	f	77,5	yes	8,19	11,79	56,85	D	2	1	150,03	452,83	33,13
cSCC	34	EE	m	80,4	yes	14,98	14,03	120,15	D	3	2	310,67	1545,00	20,11
cSCC	35	FF	m	81,9	yes	2,26	5,70	10,34	none	3	1	n.e.	n.e.	n.e.
cSCC	36	GG	f	55,3	yes	8,28	12,69	28,94	I	2	1	445,24	1489,60	29,89
cSCC	37	HH	m	84,5	no	0,00	20,33	108,74	D	3	0	106,83	1335,50	8,00
cSCC	38	II	m	76,1	yes	1,33	11,54	54,22	D	2	1	39,80	199,94	19,91
cSCC	39	JJ	m	84,1	no	0,00	18,06	100,80	D	2	1	567,43	883,09	64,26
cSCC	40	KK	f	83,3	no	0,00	20,58	112,97	G, D	3	2	865,80	1301,50	66,52
cSCC	41	LL	m	86,7	no	0,00	6,01	19,00	none	2	0	143,07	1358,10	10,53
MCC	42	MM	f	84,4	yes	25,74	13,59	116,20	n.a.	3	2	52,94	141,32	37,46
MCC	43	NN	f	84,9	yes	12,46	14,36	96,17	n.a.	2	1	979,68	486,42	201,41
MCC	44	OO	f	79,9	yes	7,32	9,96	54,91	n.a.	2	3	37,30	348,27	10,71
MCC	45	PP	m	89,0	no	0,00	18,75	129,32	n.a.	1	0	278,36	400,02	69,59
MCC	46	QQ	m	74,6	no	0,00	9,83	71,97	n.a.	1	0	41,09	567,30	7,24

MCC	47	RR	m	74,7	no	0,00	10,36	66,90	n.a.	1	0	19,50	556,26	3,51
MCC	48	SS	f	81,2	no	0,00	13,67	92,68	n.a.	0	0	79,58	379,63	20,96
MCC	49	TT	f	81,8	no	0,00	21,46	97,79	n.a.	1	1	n.e.	n.e.	n.e.
MCC	50	UU	f	89,3	no	0,00	17,69	173,63	n.a.	1	0	1,23	12,62	9,76
MCC	51	VV	m	80,8	no	0,00	9,06	22,03	n.a.	2	0	1855,80	708,96	261,76
MCC	52	WW	f	76,4	no	0,00	13,25	68,29	n.a.	1	0	185,81	34,75	534,71
MCC	53	XX	m	77,1	no	0,00	24,54	241,67	n.a.	0	0	151,98	752,96	20,18
													D = invasion depth ≥ 6mm	
													I = immunosuppression	
													G = grading ≥ G3	
													n.e. = not evaluable	
													n.a. = not applicable	
