# Supplementary material for: Evidence of Neutrophils and Neutrophil Extracellular Traps in Human NMSC with Regard to Clinical Risk Factors, Ulceration and CD8+ T Cell Infiltrate
Source: Int J Mol Sci. 2024 Oct 2;25(19):10620. doi: 10.3390/ijms251910620 (PMC11476888; doi:10.3390/ijms251910620)
Supplement: Supplementary file 1 [file ijms-25-10620-s001.zip › Table S2.pdf]

**Table S2**: Overview of metastases									
**tumor type**	**sample**	**patient**	**sex**	**age [years]**	**localization**	**ulcerated**	**necrotic**	**neutrophil score [0-3]**	**NET score [0-3]**
cSCC	54	O	m	70,51	skin	no	no	2	0
cSCC	55	YY	m	67,53	skin	no	no	1	0
cSCC	56	ZZ	m	71,30	lymph node	-	yes	3	0
cSCC	57	W	m	91,44	skin	no	yes	3	1
cSCC	58	X	m	69,98	lymph node	-	no	2	0
cSCC	59	Y	m	80,44	skin	yes	no	2	2
cSCC	60	AAA	m	74,31	skin	no	no	2	0
cSCC	61	BBB	f	51,72	skin	no	no	2	0
MCC	62	CCC	f	52,61	lymph node	-	yes	1	0
MCC	63	DDD	f	67,59	lymph node	-	yes	1	0
MCC	64	EEE	m	70,17	salivary gland	-	no	2	0
MCC	65	FFF	m	73,96	adrenal gland	-	yes	2	0
MCC	66	GGG	m	66,98	skin	no	no	1	0
